# Supplementary material for: Dynamic crystallography reveals spontaneous anisotropy in cubic GeTe
Source: Nat Mater. 2023 Feb 20;22(3):311–5. doi: 10.1038/s41563-023-01483-7 (PMC9981458; doi:10.1038/s41563-023-01483-7)
Supplement: Supplementary file 1 — Supplementary Sections 1–9 and refs. 1–39. [file 41563_2023_1483_MOESM1_ESM.pdf]

nature materials

Letter

https://doi.org/10.1038/s41563-023-01483-7

Dynamic crystallography reveals spontaneous anisotropy in cubic GeTe

In the format provided by the authors and unedited

Contents

S1: Analysis of the average structure of GeTe as a function of temperature from X-ray diffraction. 1
1.1 Results of Rietveld refinements . . . 1
1.2 The critical temperature for the R-C phase transition . . . 3
1.3 The 'standard' PDF experiment . . . 3
1.4 Results of small-box structure refinements against the X-ray PDFs of GeTe . . . 4

S2: Comparison of very high resolution synchrotron powder X-ray diffraction data for c-GeTe and Si. 5

S3: Neutron scattering theory, definition of quantities measured, extraction of PDFs, and motivation for the vs-PDF technique. 7
3.5 Measuring $S(Q,\omega)$ . . . 7
3.6 Dynamic pair distribution functions . . . 8

S4: Influence of the resolution function on the vsPDF analysis 11
4.1 Resolution function of the ARCS spectrometer . . . 12
4.2 The raw elastic line PDF of GeTe . . . 13
4.3 Use of principle component analysis to extract the elastic and inelastic PDFs . . . 13

S5: Comparison of the radial distribution functions extracted from the MD simulations with X-ray data. 14

S6: Phonon spectroscopy for GeTe. 15

S7: Real-space thermal diffuse scattering in GeTe as a function of temperature. 16

S8: Ubiquity of <100>$_c$ correlations in binary chalcogenides and hR6 structured materials. 17
8.1 The effect of correlated motion/phonons on the pair distribution function . . . 18
8.2 The state of the art for modelling correlated motion using 'small box' PDF refinements . . . 18
8.3 Published evidence for emergent anisotropy in other binary chalcogenides and hR6 structured materials . . . 19

S9: Extended theory results. 21

References. 22

S1:Analysis of the average structure of GeTe as a function of temperature from X-ray diffraction.

1.1 Results of Rietveld refinements

Our medium-resolution powder X-ray diffraction data, which were collected on ID15B at the European Synchrotron Radiation Facility, are highly consistent with literature results. As shown in Fig. **S1**, a clear rhombohedral Bragg peak splitting is observed at room temperature. This disappears at 650 K, above which a metrically cubic unit cell describes the data. Data were collected in 25 K steps on warming from room temperature.

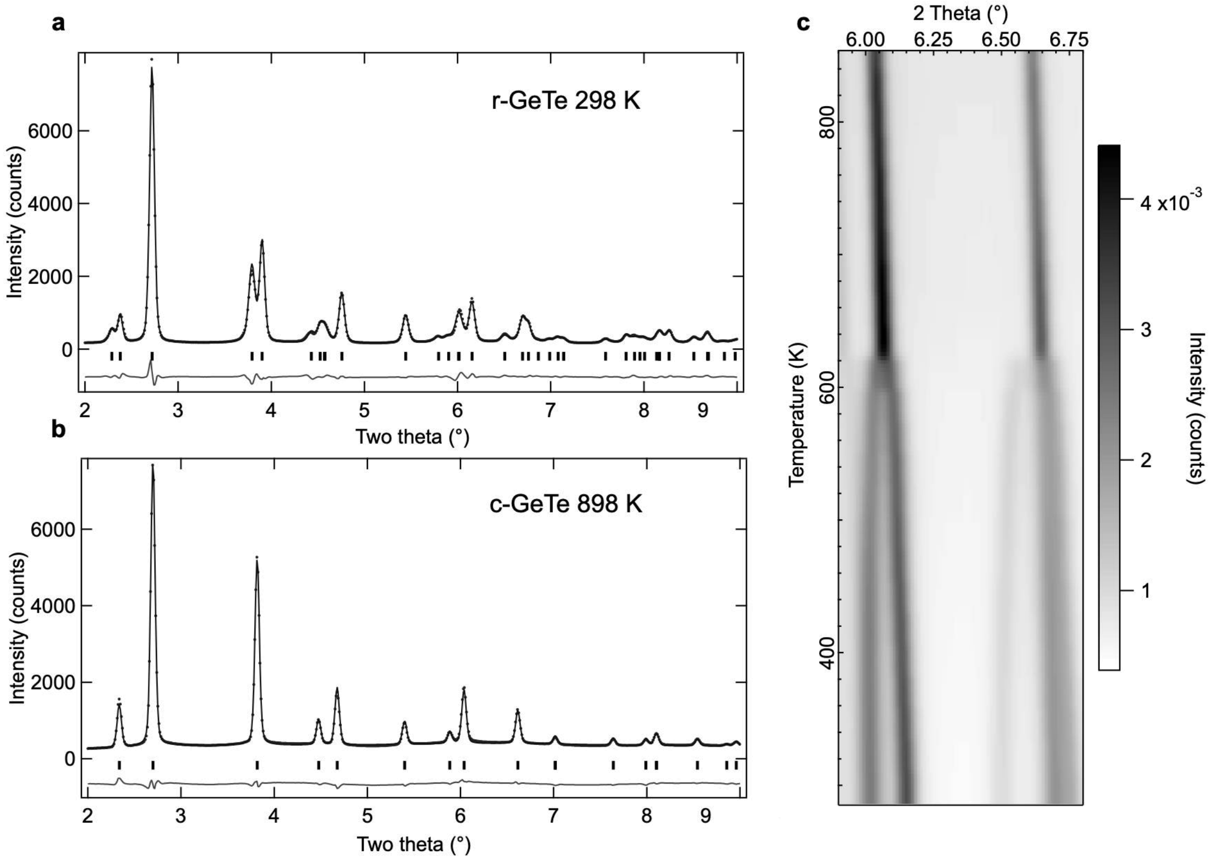

S1. 1| Results of fitting the average structure of GeTe using Rietveld refinements against synchrotron X-ray powder diffraction data. **a,** Observed, calculated and difference profiles from a portion of the fit at 298 K. A clear splitting of the fundamental Bragg peaks can be observed, reflecting the rhombohedral symmetry;**b,** Observed, calculated and difference profiles from a portion of the fit at 898 K (the highest temperature reached by us). The splitting of the fundamental Bragg peaks has disappeared, reflecting the cubic symmetry; **c,** A portion of the observed X-ray diffraction data shown as a function of temperature in the form of a map. This highlights the smooth convergence of the split Bragg peaks at 650 K.

The atomic parameters, and selected fit parameters, resulting from our Rietveld refinements are shown in Table I. The actual two theta range refined over was 1.5 < 2 theta < 12 degrees. Note that an anisotropic expansion of the peak shape was necessary to account for strain broadening in the rhombohedral phase. This effect disappears in the cubic phase within the limits of our resolution. A five term Chebyschev polynomial was used as a background in the rhombohedral phase. At higher temperatures, more terms were necessary to account for the highly structured thermal diffuse scattering present, as described in more detail in the main text. Note the rather large refined atomic displacement parameters. At high temperatures, these are more characteristic of flexible organic molecules than high-symmetry inorganic materials.

Table 1| Selected crystallographic data from the Rietveld refinements of the GeTe structure against synchrotron X-ray diffraction data.

Temperature (K)	Space Group	a (Å)	α (°)	Volume (Å^2)	B_{Ge} (Å^2)	B_{Te} (Å^2)	R_{wp} (%)
298	$R3m$	4.303(5)	58.097(8)	53.898(5)	1.22(6)	0.52(3)	4.65
898	$Fm\bar{3}m$	6.03983(16)	-	220.330(17)	5.99(4)	2.67(8)	4.45

In figure **S1.2**, we show some of the extracted refined parameters as a function of temperature. These include the reduced rhombohedral cell volume, the first-coordination sphere Ge-Te bond distances, and the thermal parameters. These results are consistent with those published elsewhere [1].

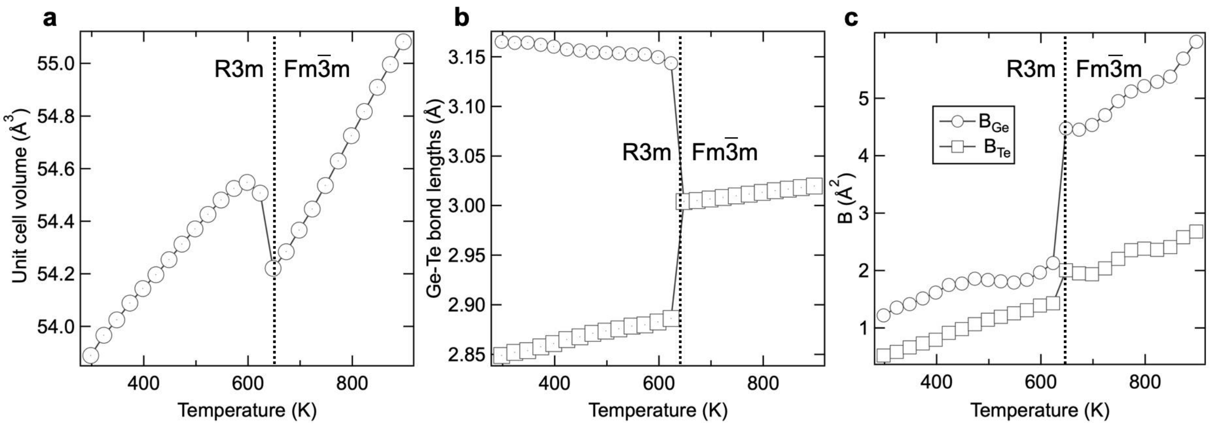

S1. 2| Temperature dependence of refined parameters from Rietveld refinements against synchrotron X-ray powder diffraction data. **a,** Temperature dependence of the refined rhombohedral unit cell of GeTe.;**b,** Refined GeTe bond distances in both phases as a function of temperature; **c,** Refined atomic displacement parameters as a function of temperature. Note the large jump in B_{Ge} at the phase transition.

1.2 The critical temperature for the R-C phase transition

Since a range of temperatures have been reported in the literature for this phase transition, we summarise the range of values for T_c in Table 2, and briefly discuss this issue.

Note that our work employed the same sample of GeTe used [2] to characterise the R-C phase transition by Chattopadhyay, Boucherle and von Schnering in 1987. This is referred to as the CBvS sample. Other samples are either: a) research grade, described as 'synthetic'; or b) commercial, as provided by e.g. Alfa Aesar.

The phase transition temperature of GeTe is known to be strongly affected by impurities, and we immediately note that the only commercial sample in the list has by far the lowest T_c of 600 K. This sample was additionally described as showing a GeO_2 impurity (and hence likely significant levels of vacancies) [1].

We further note that, with the exception of the original 1987 experiment performed on the CBvS sample, most other measurements fall in the range 640-663 K. The value of T_c for our measurement is thus consistent with other literature reports. Further investigation of this issue is clearly merited, but beyond the scope of this paper. We speculate that differences in temperature calibration, or perhaps angular resolution, may also contribute to the differences between measurements. These uncertainties are likely to be at least at important as any changes caused by defects or non-stochiometry in our opinion.

Table 2| Comparison of literature values for the rhombohedral-cubic phase transition in GeTe.

Reference	Sample	T_c	Method
This work (2022)	CBvS sample	650 K	Synchrotron X-ray powder diffraction
Chattopadhyay *et al* [2] (1987)	CBvS sample	700 K	Neutron single crystal diffraction
Levin *et al* [3] (2013)	synthetic	640 K	Synchrotron X-ray powder diffraction
Chatterji *et al* [1] (2015)	commercial	600 K	Neutron powder diffraction
Sist *et al* [4] (2018)	synthetic (?)	645 K	DSC analysis
Gainza *et al* [5] (2019)	synthetic	663 K	Synchrotron X-ray powder diffraction

1.3 The 'standard' PDF experiment

Many thousands of pair distribution functions are experimentally derived from X-ray, neutron and electron scattering on an annual basis. Indeed, this technique has become a standard characterisation tool for solid state and

liquid samples. Except for a tiny number of experiments, these are *energy integrated* measurements. That it is to say, no energy analysis of the scattered beam is performed. These measurements are thus only valid if the energy of the incident beam far exceeds that of the typical fluctuations in the system (the quasi-static approximation). In the case of an X-ray scattering measurement this is clearly valid, since the characteristic energy of the beam (keV) far exceeds that of lattice vibrations ($\sim$0.1 eV), not to mention the over-damped fluctuations in liquids or e.g. ionic conductors. This is not necessarily always true in the case of neutron scattering e.g. for magnetic diffuse scattering [6], since the trajectory of a low-angle (2θ) detector is strongly curved in wavevector (**Q**) and energy (ω) space, due to the kinematic condition described later. When correctly performed, such energy integrating measurements yield the scattering function S(**Q**):

$$S(\mathbf{Q}) = \int_{-\infty}^{\infty} S(\mathbf{Q},\omega) d\omega = \frac{1}{N\langle b \rangle^2} \sum_{\upsilon,\mu} b_\upsilon b_\mu \left\langle \left\langle e^{i\mathbf{Q}\cdot(\mathbf{R}_\upsilon(0)-\mathbf{R}_\mu(0))} \right\rangle \right\rangle \tag{1}$$

Here the pair correlations between atoms $\mathbf{R}_\upsilon$ and $\mathbf{R}_\mu$ are weighted by the respective neutron scattering lengths, b_υ and b_μ or by the relevant X-ray form factors. This expression only contains $t = 0$ terms, and thus the *instantaneous* atomic correlations only. A real experiment (during which the sample is exposed to the beam for a long time relative to atomic motion), may hence be regarded as the sum of all of the possible snap-shot configurations explored at a given temperature. In general, the local distribution of positions is not the same as the average long-range positions probed by e.g. diffraction techniques.

The pair distribution function may be recovered from an experimental S(Q) by properly normalising the observed signal as described elsewhere [7] and later in the **SI**. The pair distribution structure factor, $F(Q)$ is first calculated as follows:

$$F(Q) = Q.[S(Q) - 1] \tag{2}$$

A sine Fourier transform then yields the familiar pair distribution function $G(r)$:

$$G(r) = \left(\frac{1}{2}\right) \int_{Q_{min}}^{Q_{max}} F(Q)\sin(Qr) dQ \tag{3}$$

In the work reported here, the so-called 'small box' method [8] is used for model fitting the observed PDFs. A model calculation, $G_{calc}(r)$, (which uses unit cell based symmetry constraints) is calculated and refined against the data, $G_{obs}(r)$ using least squares methods. This minimised the residual $G_{diff}(r) = G_{obs}(r) - G_{calc}(r)$. The master equation (4), which is a double sum over all atoms in the unit cell, is shown below. For neutron PDF data, the contribution of the different distances is weighted by the appropriate scattering lengths, b_i. In the case of X-ray scattering data, the scattering lengths, b_i, are replaced by the value of the X-ray form factor at Q=0 for each species, i.e. $f(Q = 0)$.

$$G_c(r) = \frac{1}{r} \sum_i \sum_{j \neq i} \left[\frac{b_i b_j}{\langle b \rangle^2} \delta(r - r_{ij}) \right] - 4\pi r \rho_0 \tag{4}$$

1.4 Results of small-box structure refinements against the X-ray PDFs of GeTe

In the section, we describe our strategy for fitting the X-ray PDFs of GeTe, and show selected results. Our room temperature (298 K) and highest temperature (898 K) data are shown in Fig. **S1.3**. The pair distribution function structure factors, $F(Q)$ (which are normalised and corrected for the X-ray form factors) are shown in panels **S1.3a** and **S1.3b**. This is the raw quantity which is Fourier transformed to yield the PDF. Of particular interest is panel **S1.3b** at 898 K. This can be seen to be extremely similar to the neutron $F(Q)$ shown in the main text in Fig. 2a. Normally, one would expect significant differences between X-ray and neutron data sets. However, $F_{XRD}(Q)$ is already corrected for the form factors, and secondly the intensity of the (odd Miller indice) superstructure reflections in the NaCl structure is proportional to the difference in scattering power of the two species. This is of comparable magnitude for the neutron and X-ray scattering cases (b_{Ge}/b_{Te} $\sim$1.4 and Z_{Te}^2/Z_{Ge}^2 $\sim$2.64). Of particular note, is that a high-Q diffuse signal similar to the oscillation reported in the main text is also seen (inset **S1.3b**). This is weaker in the X-ray case (likely confirming that it relates to Ge atomic motion) and can also be fitted as $\sim \sin(Q.r)$ with r = 2.87(6) Å.

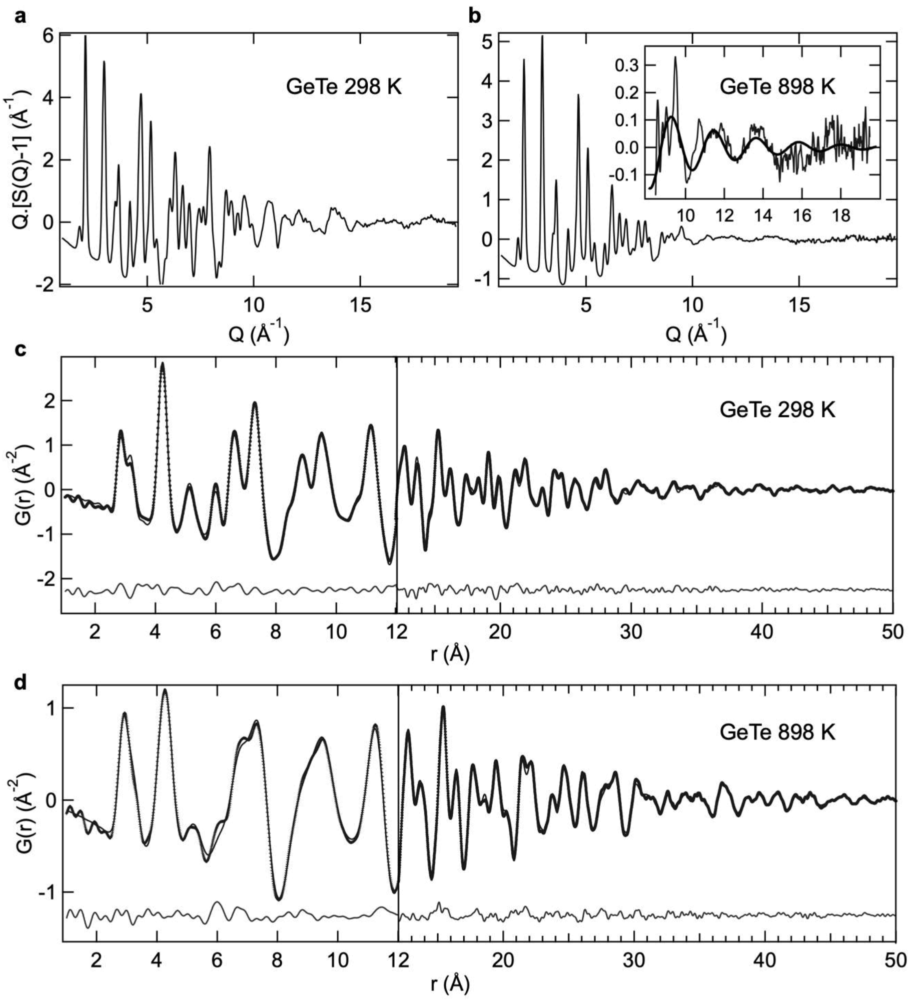

S1. 3| Total scattering data and fits to the pair distribution function of GeTe. a, The reciprocal space structure factor $F(Q) = Q.[S(Q) - 1]$ for GeTe at 298 K; **b,** The reciprocal space structure factor $F(Q) = Q.[S(Q) - 1]$ for GeTe at 898 K in the cubic phase. Similar to the result shown in Fig. 2a of the main paper, an oscillatory diffuse component is seen at high-Q; **c,** Composite fit to the pair distribution function of GeTe at 298 K (ranges are 1 < r < 12 Å and 12 < r 50 Å); **d,** Composite fit to the pair distribution function of GeTe at 898 K (ranges are 1 < r < 12 Å and 12 < r 50 Å).

Fits were performed over varying ranges of r_{min} < r < r_{max}, which gives access to the structure on varying length scales. Below the structural phase transition, the PDFs were fitted using the trigonal average structure. In addition to crystallographic variables, we refined an overall scale, a parameter which accounts for resolution effects (Q_{damp}), and a 'sharpening parameter' to account for correlated motion. These are all described in standard works [7, 9], and a more detailed explanation [10, 11] of the latter term can be found in section **S3.2**. At room temperature, the refined parameters were: a=4.3015 Å; α ==58.20°; x_{Ge}=0.531; B_{Ge}=2.37 Å^2; B_{Te}=1.44 Å^2. At higher temperatures (above 650 K), the average structure is metrically cubic, yet the $Fm\overline{3}m$ structure fails to fit the asymmetric first coordination sphere peak at ~3 Å. We therefore introduced disorder with an eight-fold splitting of the Ge site. This transformed the original fully occupied special position $4a$ site on ($\frac{1}{2}$,$\frac{1}{2}$,$\frac{1}{2}$) into an 1/8 occupied general 32-fold site on (x, x, x). At 898 K, the fit shown converged with: a=4.3015 Å; B_{Ge}=4.58 Å^2; B_{Te}=3.11; x_{Ge}=0.526.

S2: Comparison of very high resolution synchrotron powder X-ray diffraction data for c-GeTe and Si.

The shape and width of the peaks in a powder diffraction pattern can indicate the presence of defects and disorder. Provided that the contribution of the instrument resolution function is small, these manifest as extra 'microstrain' broadening. The data shown in Fig. **S2a** for c-GeTe were collected at 673 K on the highest resolution X-ray diffractometer in the world (then ID31 at the European Synchrotron Radiation Facility). The absence of any significant broadening (compared to a Si line width reference, Fig. **S2b**) support our conclusion that static nanoscale inhomogenieties are absent in c-GeTe. Both data sets were collected in capillary geometry with an incident X-ray energy of $\sim$30 KeV.
The inset to Fig. **S2a** shows the strongly featured thermal diffuse scattering which accompanies the Bragg reflections. At the low-Q region highlighted here, this is strongly peaked under the peaks, as expected for acoustic phonons. Note how this signal can contaminate the structure factors extracted by Rietveld refinement, if not properly corrected for [12, 13].

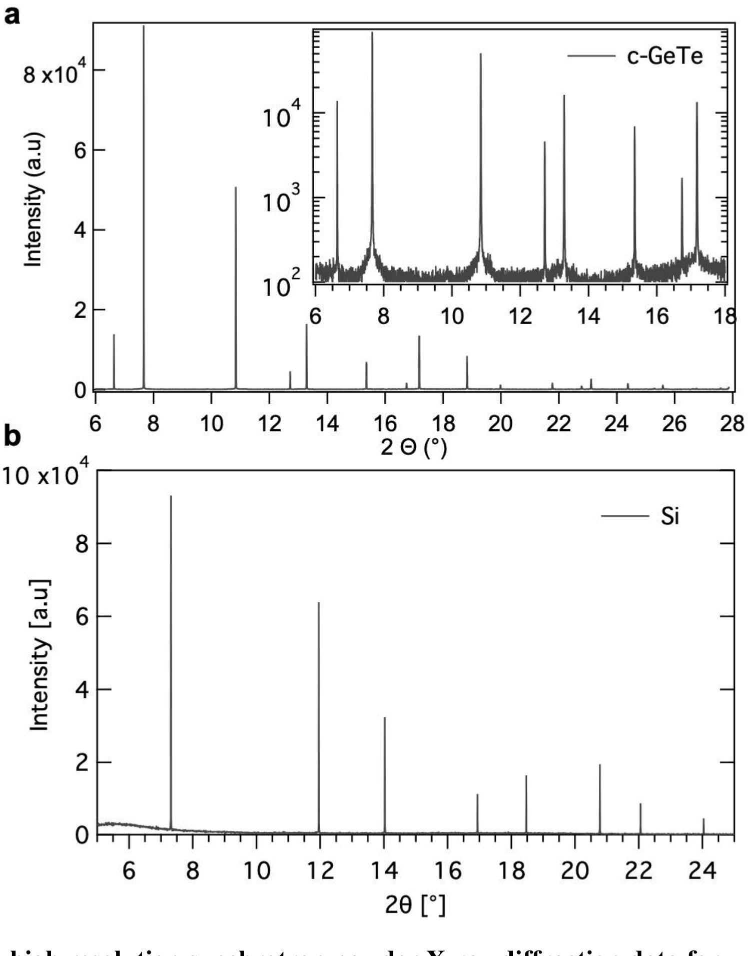

S 2| Comparison of very high resolution synchrotron powder X-ray diffraction data for c-GeTe and Si. Data were collected on the ID31 instrument at the European Synchrotron Radiation Facility, the highest resolution instrument in the world at the time. Silicon is used as a line shape reference due to its essentially perfect lattice coherence. The peaks from c-GeTe are nearly as sharp, showing that this phase is highly crystalline. Note the log scale and the co-existence of structured diffuse scattering shown in the inset for c-GeTe. The featureless background on the Si data is more intense due to a shorter counting time.

S3: Neutron scattering theory, definition of quantities measured, extraction of PDFs, and motivation for the vs-PDF technique.

This part of the **SI** gives a brief introduction to the quantities measured using neutron and X-ray scattering, and the role of energy resolution. Following standard works [7, 14], we focus on the neutron case, vector quantities are denoted in bold, and $\langle\rangle$ denotes an ensemble average.

A neutron detector of area a, covering solid angle Ω, measures the double differential cross section (5), where σ is the total sample cross section, and E_i and E_f are the incident and final neutron wave vectors respectively.

$$\frac{d^2\sigma}{d\Omega d\omega} = a^2 \left(\frac{E_f}{E_i}\right)^{1/2} S(\mathbf{Q},\omega) \tag{5}$$

The quantity $S(\mathbf{Q},\omega)$ is the *scattering function* (11), which describes the scattered neutron intensity from the sample as a function of wave vector ($\mathbf{Q} = 4\pi \sin\theta/\lambda$) and energy transfer ($\omega$). In our work, the units of the latter are always given in meV.

$$S(\mathbf{Q},\omega) \equiv \frac{1}{2\pi}\int_{-\infty}^{\infty} F(\mathbf{Q},t)e^{i\omega t}dt \tag{6}$$

By Fourier transform in the energy domain, $S(\mathbf{Q},\omega)$ yields the *intermediate scattering function*, $F(\mathbf{Q},t)$ (7). Note that the similar notation does not imply any relation to the powder averaged pair distribution structure factor F(Q), which was introduced earlier.

$$F(\mathbf{Q},t) \equiv \int G(\mathbf{r},t)e^{-i\mathbf{Q}\cdot\mathbf{r}}d\mathbf{r} \tag{7}$$

Finally, $F(\mathbf{Q},t)$ is then directly related to the famous Van Hove correlation function (8) by Fourier transform in the wave vector domain. This characterises the spatial and time dependence of real-space pair correlations in the system.

$$G(\mathbf{r},t) = \left\langle \frac{1}{N}\int \sum_{i=1}^{N}\sum_{j=1}^{N} \delta[\mathbf{r}' + \mathbf{r} - \mathbf{r}_j(t)]\delta[\mathbf{r}' - \mathbf{r}_i(0)]d\mathbf{r}' \right\rangle \tag{8}$$

We can therefore express (9) the scattering function recovered by an ideal experiment in terms of the atomic positions as a function of time $\mathbf{r}_j(t)$ as:

$$S(\mathbf{Q},\omega) = \frac{1}{2\pi N}\sum_{j,j'}\int \left\langle \exp(i\mathbf{Q}\cdot[\mathbf{r}_j(0) - \mathbf{r}'_j(t)])\right\rangle \exp(i\omega t)dt \tag{9}$$

3.5 Measuring $S(Q,\omega)$

In this work, we collected inelastically resolved neutron scattering data using the ARCS spectrometer [15] at the Spallation Neutron Source, ORNL. This instrument uses a pulsed neutron beam, which is monochromated using a Fermi chopper. Since the incident energy and the instrument geometry are known, the trajectory of each detector pixel in $S(Q,\omega)$ can be calculated [note that for powder samples, only $|\mathbf{Q}|$=Q is measured]. This conversion is performed using standard macros, and uses the kinematic condition below. Note that ARCS actually has a 2D area detector, and has many pixels at each 2θ value, which are azimuthally integrated during data reduction:

$$\frac{\hbar Q^2}{2m} = E_i + E_f - 2\sqrt{E_iE_f}\cos 2\theta \tag{10}$$

Our sample of GeTe was placed in a vanadium can, and data were collected at a range of incident energies, E_i =40, 120 and 300 meV and at temperatures of 300, 550 and 720 K. This provides data suitable for both classical determination of the phonon density of states, and Fourier transformation to real space. For comparison to standard angle resolved diffractometers, these incident energies convert to neutron wavelengths of 1.43, 0.83 and 0.52 Å respectively.

We first normalised the detector solid angle and efficiencies to a white beam vanadium measurement, performed

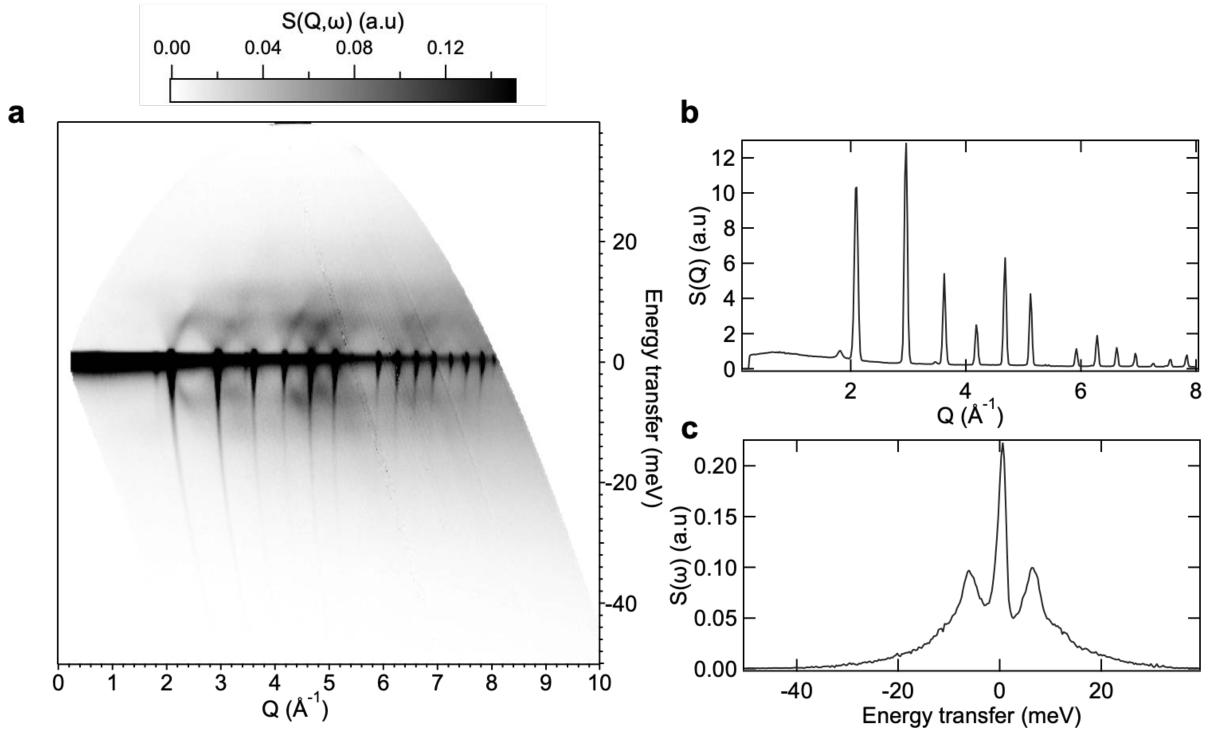

S3. 1| **a,** Example data collected using ARCS and an incident energy of 40 meV; **b,** A slice through the elastic line is shown at zero (elastic) energy transfer; **c,** A slice through the elastic line, avoiding Bragg peaks, shows phonon creation and annihilation peaks.

outside the sample environment. We then used a monochromatic E_i =300 meV vanadium measurement to correct for transmission through the MICAS-III furnace [16]. We measured an empty vanadium can at 300 and 720 K for a background, and test measurements using a B_4C mask in place of the sample showed that scattering of the incident beam from the sample environment were efficiently removed by the radial oscillating collimator [17]. The background and absorption corrections were performed using the Paalman-Pings macro implemented in MANTID [18, 19]. Finally, the spectrometer has two small detector gaps at high angle. These can be removed by extrapolation in S(Q,ω) space, as their trajectories are curved, and the signal at high-Q is relatively flat.

Example data from ARCS is shown in Fig. **S3.1**, illustrating: 1) The characteristic 'kinematic window' of accessible data, which is determined by Eqn. (10) and the detector angles; 2) A 'Q-cut' through the elastic line, highlighting the creation and annihilation of a phonon excitation on the neutron energy loss and gain side respectively; 3) An 'energy slice' through the elastic line, note how the observed intensity tends to zero at high-Q due to the Debye-Waller factor; 4) The energy resolution function of ARCS is somewhat asymmetric. In this work, we used only the neutron energy loss data. This is justified at high temperature, since $S(Q,-\omega) = \exp(-\hbar/k_bT)S(Q,\omega)$. At the energy of the crucial optical modes in c-GeTe, there is therefore an ∼91 % intensity balance between the energy-gain and energy-loss sides of $S(Q,\omega)$.

3.6 Dynamic pair distribution functions

The possibility of measuring energy resolved PDFs has been discussed for some years [10, 20, 21]. This is easiest to perform with time-of-flight neutron chopper spectrometers, although some pioneering experiments on liquids have also used inelastic X-ray spectrometers [22]. In principle, the purely elastic scattering signal, $S(\mathbf{Q},0)$, (11), should recover only the infinite time average structure correlations ($G(r,t=\infty)$), in contrast to Eqn. 9. Note that this is similar, but not identical to the average structure extracted by diffraction. This is because the information in the PDF is model independent, unlike diffraction, which relies upon various assumptions (structure model, peak shape function, thermal motion models, background modelling etc). Finally, crystallographic methods typically probe a much smaller range of reciprocal ($\mathbf{Q}$) space. In our opinion, relying upon e.g. Rietveld refinement against

medium-resolution data is therefore risky for soft, anharmonic materials like GeTe, where the thermal diffuse scattering is strongly peaked under the Bragg reflections [12, 13].

$$S(\mathbf{Q},0) = \frac{1}{N\langle b\rangle^2}\sum_{\upsilon,\mu} b_\upsilon b_\mu \left\langle\left\langle e^{i\mathbf{Q}\cdot\mathbf{R}_\upsilon}\right\rangle\right\rangle\left\langle\left\langle e^{-i\mathbf{Q}\cdot\mathbf{R}_\mu}\right\rangle\right\rangle \tag{11}$$

The variable shutter pair distribution function method (vsPDF): Several equally valid approaches to the dynamic pair distribution function can be found in the literature. The first is simply to collect an S(Q,ω) map using a direct geometry neutron spectrometer, which is then divided into slices at fixed energy transfer. These are then individually Fourier transformed. The second approach (which has very recently become prominent for liquids studied by inelastic X-rays) involves a 2D Fourier transform to directly recover the Van Hove correlation function (8). This is perhaps best suited to cases where the intrinsic width of the experimental signal is similar in the Q and energy domains, and not discussed further here.

In this work, we have chosen to develop the simpler vsPDF technique, where an energy window, $0 < E_{max} < \infty$ is used. This always contains the $E = 0$ meV elastic line. Our choice was motivated by the technical and philosophical points below, and also by the prospect of routine use by the materials chemistry community in the future. These key points were identified at the *Advanced Fourier Techniques* workshop organised by SAJK at the Shull-Wollan centre (ORNL, 2017):

1. The central materials question to be answered is: Static or dynamic? What are the origins of the 'local structure' seen by pair distribution function measurements?
   This motivates a comparatively simple technique, using energy windows. The vsPDF method interpolates between two well understood limits, $G(r, t = 0)$ and $G(r, t = \infty)$.

2. The main advantage of real-space techniques is that local-structure details are model independent, and visible by inspection.
   Fourier transforming a slice at fixed energy, which only contains an inelastic signal, produces a complex 'modification function', not directly interpretable in real-space. Summing this with the elastic line PDF (as in vsPDF) makes the effect upon atomic correlations clear, preserving the main advantage of real-space techniques.

3. Correctly normalising data is crucial to avoid artefacts from Fourier transformation. What is the best way to do this for slices through S(Q,ω) space, and to constrain the final result?
   As discussed below, cuts at fixed energy (including at the elastic line) do not tend to well-behaved limits for Fourier transformation, requiring normalisation. By summing over $0 < E_{max} < \infty$, the normalisation of each energy resolved PDF is constrained by the (strong) elastic signal.

4. How to deal with the interplay between Q-space and energy resolution which is intrinsic to direct chopper spectrometers?
   *The Q-space and energy resolutions of direct geometry instruments is inversely correlated, is weakly dependent on energy transfer, and sometimes asymmetric. In addition, the kinematic condition imposes a varying Q_{min} and Q_{max} as a function of energy transfer. While more complex methods of interpolation and joining data sets exist, we have used principle component analysis (*vide infra*) to ameliorate this for the simple case of GeTe. We also identify developing methods, such as the super-resolution technique for the future.*

Notes on normalising vsPDF data: If the Fourier transform defined above (3) is to produce a well-behaved PDF, then the structure factor, $F(Q)$, (2) needs to oscillate around, and tend towards zero in the high-Q limit. For standard energy-integrated PDF measurements, this is achieved by assuming that the observed intensity, $I(\mathbf{Q})$, is

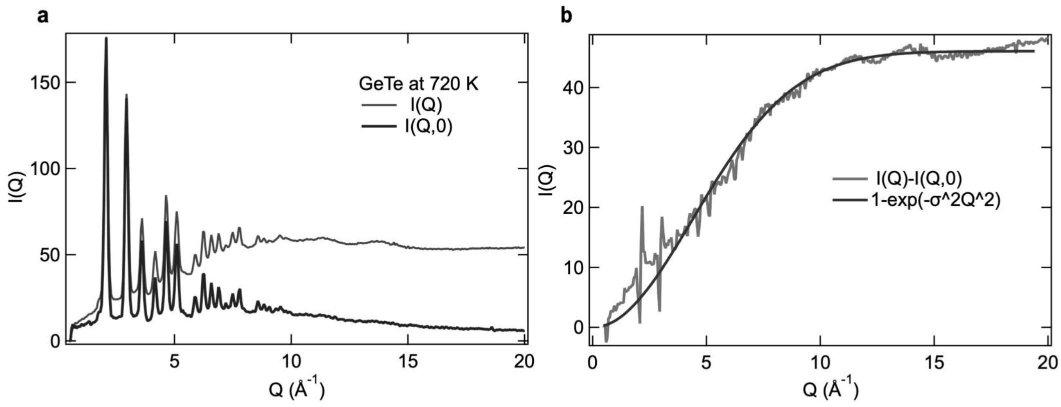

S3. 2| Example data collected using ARCS for GeTe at 720 K, with an incident energy of 300 meV. The total sum over all energies, $I(Q)$, and a cut through the elastic line, $I(Q, 0)$ are shown.

given by:

$$I(\mathbf{Q}) = \frac{d\sigma}{d\Omega}\langle b\rangle^2 - \langle b^2\rangle \tag{12}$$

And defining $S(\mathbf{Q})$ as:

$$S(\mathbf{Q}) = \frac{I(\mathbf{Q})}{\langle b\rangle^2} \tag{13}$$

Unfortunately, this normalisation breaks down when energy-resolved data is collected for samples with strong dynamics. This is because the elastic and inelastic contributions to the total neutron scattering signal have different Q-dependencies. We ignore the contribution from *static* disorder in this discussion, since the absence of this in GeTe is one of the central results of our work.

This can be seen by examining the Q-dependence of the observed total scattering, $I(Q)$, and the elastic scattering, $I(Q, 0)$ at high temperature. Here we use 'elastic' to denote the signal which can be experimentally separated within the energy resolution available at a given incident energy. Data collected for GeTe at 720 K and E_i = 300 meV are shown in Fig. **S3.2**. The changing high-Q limits as a function of energy resolution can clearly be seen (panel **a**).

The elastic intensity, $S_{elas}(\mathbf{Q}) \to 0$ at high-Q, since it is multiplied by the Debye-Waller factor, $\propto \exp^{-\frac{1}{2}\sigma^2 Q^2}$. Similarly, *individual* energy slices at finite transfer also behave similarly, since the characteristic $\propto Q^2$ phonon intensity is also damped by the Debye-Waller factor.

Fortunately, it can be shown [7] that the sum of the *total* phonon signal, which accounts for single and multiphonon processes, should tends towards:

$$S_{inel}(\mathbf{Q}) \approx 1 - \exp^{-\frac{1}{2}\sigma^2 Q^2} \tag{14}$$

Indeed, the difference between $I(Q)$ and $I(Q, 0)$ is well reproduced (panel **S3.2b**) by Eqn. (14), The oscillations around this smooth curve originate from phonon scattering (sharp features at low-Q are glitches from Bragg peak subtraction).

This result shows why the energy-integrated total scattering signal tends to a well defined constant limit at high-Q. Intensity is lost from the elastic scattering due to incoherent and coherent motions. However, this is simply shifted to higher energies, appearing as a broad background increasing with Q. The data shown in Fig. **S3.2** replies upon a very crude energy window (the resolution, $\Delta E \sim$ 14 meV), which makes it impossible to separate low-energy acoustic phonon scattering. We therefore next perform a more detailed check, which also acts as an independent verification of one of our key results. We used data collected at $E_i = 40, 120$ and 300 meV to construct a composite, high-energy resolution phonon structure factor. The inelastic signal at each E_i was separated out within the energy resolution, and stitched together as shown in Fig. **S3.3**. Again (panel **S4.3a**), $I_{inel}(Q)$ closely follows Eqn. (14), and in addition, we show the results of normalising the data by a smooth function (panel **S3.3b**), and calculating the structure factor $F(Q) = Q.[S(Q) - 1]$. This is compared to the $F(Q)$ from the total $I(Q)$, and the inelastic contribution can be seen to be independently confirmed to be the cause of the high-Q oscillations

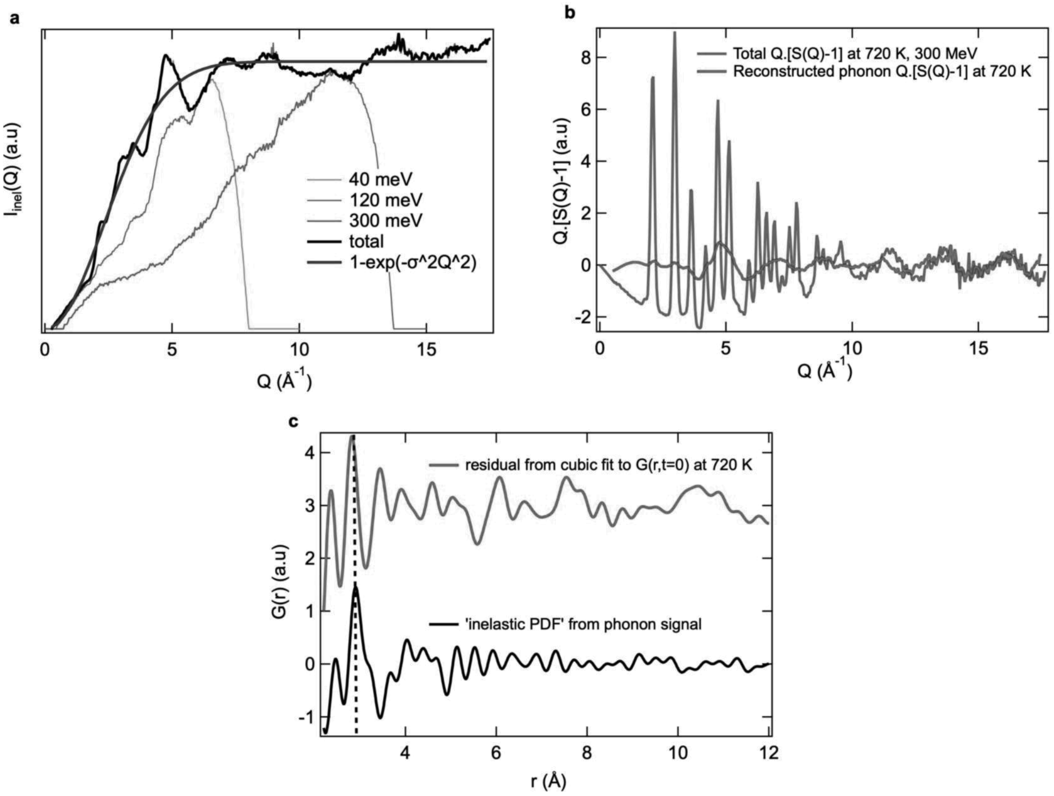

S3. 3| Example data collected using ARCS and an incident energy of 40 meV. A slice through the elastic line is shown at fixed-Q and fixed (elastic) energy transfer.

reported in Fig. 2a of the main paper. Furthermore, Fourier transformation of this signal to real-space (panel **c**) reproduces the intense peak at ~ 2.88 Å. This can be seen by the comparison to the residual, $G_{obs}(r) - G_{calc}(r)$, left after fitting the cubic GeTe model to the instantaneous PDF, $G(r, t = 0)$ (also taken from Fig. 2 of the main paper). Finally, the results in this section show how to proceed with normalising vsPDF data collected as a function of E_{max}. These can simply be normalised to a smooth function like Eqn. 14, since it contributes no structure at the r-range of interest in real space. In our work this was performed using PDFGetN3 [23].

S4: Influence of the resolution function on the vsPDF analysis.

4.1 Resolution function of the ARCS spectrometer

The resolution of time-of-flight neutron spectrometers is fairly complex, depending upon incident energy, scattering angle and energy transfer. However, the Q-space and energy-space resolutions can be decoupled to first order. Examining Q-resolution first, we fitted the peak widths of GeTe (which are resolution limited at all temperature) as a function of Q at all three incident energies (40, 120, 300 meV). As expected (**S4.1a**), these show only a very weak angular dependence, and follow the expected order (i.e. $\Delta Q_{40meV}/Q < \Delta Q_{120meV}/Q < \Delta Q_{300meV}/Q$).
We now briefly comment upon the manifestation of Q-resolution in the pair distribution function. In real space, this corresponds to a slow damping as a function of r, and therefore has no effect upon the local structure examined in our work. In the small box models employed here, we refined a parameter `Qdamp`, which generates a corresponding Gaussian damping, as described elsewhere [7, 9]. This was first refined over a large r-range, then fixed for the local fits shown in the main text.
The energy resolution of each configuration was calculated in the Carpenter approximation [24] for time of flight instrumentation, and is shown for E_i = 120 and 300 meV in Fig. **S4.1b**. This can be seen to be weakly dependent on energy transfer. Note that approximately doubling the incident energy reduces the energy resolution by more than a factor of three.
In Fig. **4.1c**, we show selected data from the elastic line measurements with E_i = 40 meV. These show the characteristic peak splitting of r-GeTe disappearing, confirming that our highest temperature measurements are indeed in the cubic phase.

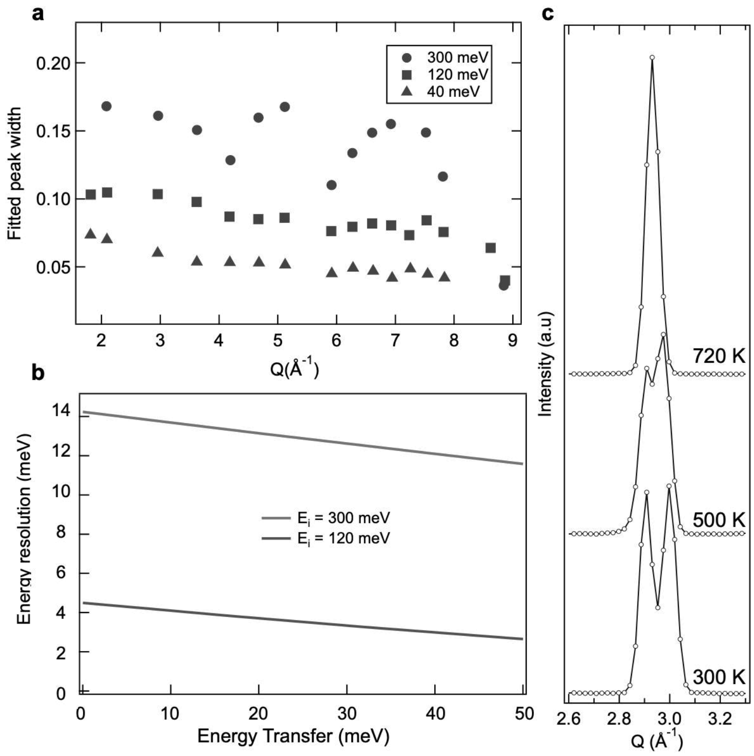

S4. 1| Resolution function of the ARCS spectrometer. **a,** We extracted the experimental Q-resolution of the spectrometer using Gaussian peak fits to the GeTe elastic line data. Note that these peak widths are resolution limited at all temperatures. The increased scattering for the E_i = 300 meV measurements comes from peak overlap; **b,** The energy resolution function of ARCS was calculated for E_i=120 and 300 meV using the Carpenter approximation for time of flight instrumentation, as implemented by Doug Abernathy (details on request); **c,** Experimental elastic line data at all three sample temperatures on ARCS with E_i = 40 meV. A clear transition from rhombohedral-cubic symmetry can be seen, confirming that the neutron sample underwent the phase transition.

4.2 The raw elastic line PDF of GeTe

In this section, we examine the effect of a simple energy window on the PDF of c-GeTe at 720 K. The data shown here (Fig. **S4.2**) we generated by extracting a slice from the raw $S(Q, \omega)$ map at zero energy transfer, and using PDFGetX3N to convert the data to real space. A cubic NaCl structure model was refined using the small-box approximation in PDFGui [8]. The first coordination sphere distortion is strongly reduced, however, a small ad-mixture of inelastically scattered neutrons is still present due to the finite 14 meV energy resolution. This motivated us to explore the more sophisticated solution to energy resolution described below.

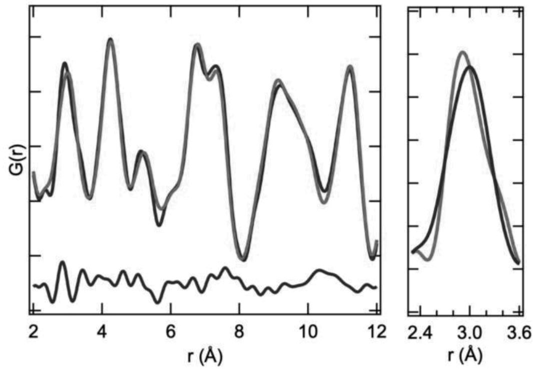

S4. 2| Fit to elastic line PDF of c-GeTe at 720 K.Observed, calculated and difference profiles for the fit of the NaCl structure to the elastic line PDF at 720 K. Note that a small ad-mixture of inelastically scattered neutrons is still present due to the finite 14 meV energy resolution. This results in a trace of first-coordination sphere distortion.

4.3 Use of principle component analysis to extract the elastic and inelastic PDFs

The results in the previous section show that a crude energy window nearly entirely removes any apparent local structure features in GeTe, and that e.g. the first coordination sphere splitting and <100>$_c$ sharpening are dynamic in origin. While this energy resolution can be strongly improved by using a lower E_i, this also sacrifices real-space resolution, by reducing the Q_{max} limit in Eqn. 3.

In order to go further, we employed principle component analysis (PCA) to better separate the elastic and inelastic scattering. This technique has been successfully employed to analyse PDF data collected on chemically transforming samples as a function of time [25, 26]. This approach work in the case of GeTe for two reasons: 1) We have multiple incident energies available; 2) Anharmonicity is so strong in the c-GeTe, that (at least with rather high E_i), the inelastic signal is very broad and almost featureless in energy.

Our strategy for the PCA analysis at 720 K was as follows: 1) We assumed that the only difference between the E_i =120 meV and 300 meV data was their resolution functions; 2) We assumed that the weights of components extracted from the E_i =120 meV data could be used to reconstruct the 300 meV data with higher energy resolution, while retaining the advantage of a large Q_{max}; We applied the PCA method to real-space PDFs calculated as a function of E_{max}.

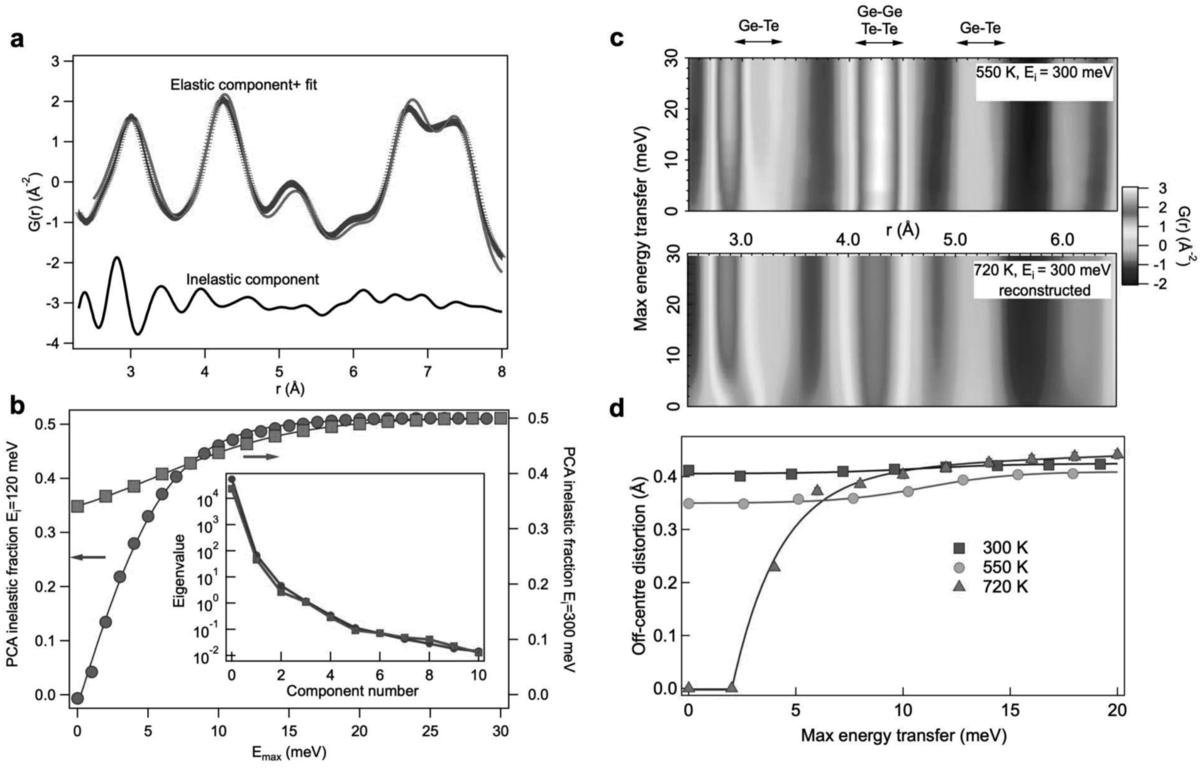

S4. 3| Details of the principle component analysis applied to the dynamic PDF data. **a,** Applying PCA analysis to the dy-PDF data sets at 720 K generated two significant Eigenvalues. As shown in the top panel, these correspond to the elastic line PDF, and the inelastic modification function. The former has been overlaid with a calculation for rocksalt GeTe with no peak sharpening factor applied. Note the similarity of the inelastic component to the fit residual for the instantaneous PDF shown in Fig. 2. **b,** The separation between the two components was determined by linear combination for the 120 and 300 meV data sets. Both show the same trend, but the former (with much higher energy resolution), allows complete separation.**c,** Reconstructed PDFs using the weights from the 120 meV data applied to the 300 meV data at 550 and 720 K. **d,** Results of fitting the first coordination sphere using a two-Gaussian approach. Note the weak shift seen in the rhombohedral phase. In the cubic phase, a single peak was fitted when the position error from a two Gauss fit was greater than the refined peak splitting.

The PCA analysis was performed in the Igor Pro software, with the aid of the included PCA demo. This can be found in: `File Menu>Example Experiments>Analysis>PCA demo`. The input data were organised in 2D matrices as a function of E_{max} and r-space. Both the 120 meV and 300 meV data sets were discovered to have two main components. Examination of these (Fig. **S4.3a**) was strongly reminiscent of the calculated cubic average structure PDF of GeTe, and the unfitted residual signal. As expected, the components were much better separated in the 120 meV data, due to the higher energy resolution (Fig. **S4.3b**). We were then able to use the weights extracted from this data set to reconstruct the E_i = 300 meV data (Fig. **S4.3c**), resulting in the essentially perfect cubic elastic PDF shown in the main paper (Fig. 2b). This data set also allowed us to determine the energy scale for the dynamic features in the data. as shown in Fig. **S4.3d**, for the first coordination sphere.

The refinement of the NaCl structure in Fig. 2b was performed in space group Fm$\overline{3}$m, with Te on (0,0,0) and Ge on ($\frac{1}{2}$,$\frac{1}{2}$,$\frac{1}{2}$). This converged with a=6.055 Å, B_{Ge}=1.68 Å^2, B_{Te}=4.7 Å^2. A reasonable value of δ_2=2.65 was refined, presumbaly reflecting the isotropic effect of low-energy acoustic phonons. An arbitrary scale was refined, and Q_{damp}=0.071 was determined using a fit over a wide r-range, and fixed here.

To conclude this section, we note that more general tools also exist, which might be fruitfully applied to vsPDF data. These include e.g. non-negative matrix factorisation [27, 28] and super-resolution techniques [29].

S5: Comparison of the radial distribution functions extracted from the MD simulations with X-ray data.

We used the trajectories from the *ab-initio* molecular dynamics simulation to calculate a radial distribution function (RDF) for comparison with experiment. The RDF was weighted with the appropriate scattering factors, i.e. the intensity from a vector between atom i and atom j is $\propto Z_i Z_j$, where Z_i represents the value of the X-ray form factor at $Q = 0$ for atom i. The RDF was calculated for each step in the trajectory, then averaged.
As seen in Fig. **S5**, the agreement with experiment at low-r is excellent. At higher-r, finite size effects become more important.

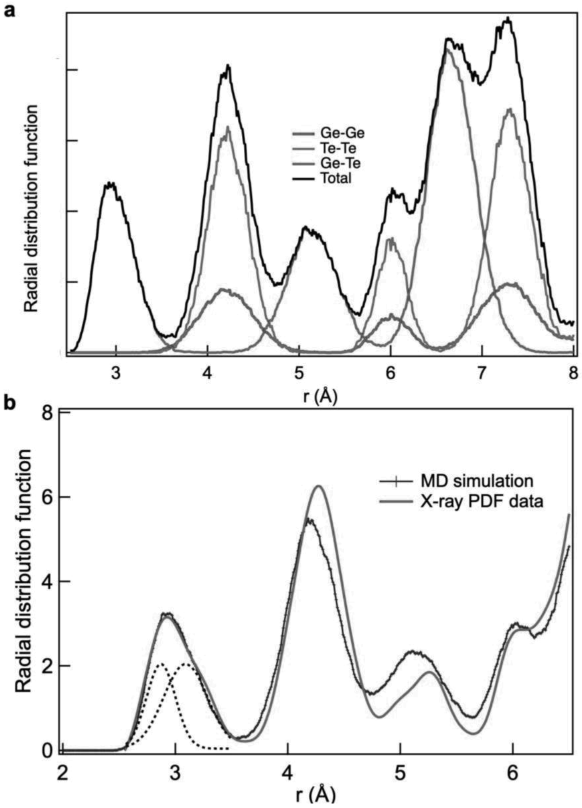

S 5| Calculated X-ray weighted radial distribution functions from MD simulations, and comparison to X-ray PDF data.a, partial PDFs for c-GeTe from our *ab initio* MD simulation at 720 K. **b** Comparison of the total X-ray weighted radial distribution function to experimental data at 720 K. Note the excellent agreement between simulation and data in the first coordination sphere.

S6: Phonon spectroscopy for GeTe.

We used the ARCS data collected using E_i =40 meV to calculate the phonon density of states (PDOS) of GeTe, and to examine the powder-averaged phonon dispersion in reciprocal space. Our results for the PDOS are in excellent agreement with literature reports [30], as shown in Fig. **S6a**. The PDOS of GeTe changes significantly with temperature, largely due to a collapse and broadening of the optical phonons.
We also show the powder-averaged $S(Q, \omega)$, which we calculated using the phonon frequencies and polarisation vectors extracted from the *ab-initio* molecular dynamics simulations. When compared to experiment (Figs. **S6b** and **S6c**), this result highlights the massive anharmonicity in c-GeTe. The acoustic phonons are well captured by the harmonic simulation, however, a sharp band of intensity is seen around 11.5 meV, which is missing in experimental data. This corresponds to the turning points of the optical phonon dispersion shown in the main text (Fig. 3b). This signal is entirely washed out in the experiment, with intensity redistributed over a large area of $S(Q, \omega)$. This highlights one of the unique advantages of Fourier techniques like the pair distribution function.

These are excellent at capturing diffuse elastic or inelastic scattering, and repackaging it as features at low-r. In contrast, these signals can be difficult to capture using reciprocal space techniques, especially those which measure only limited areas of $S(Q,\omega)$.

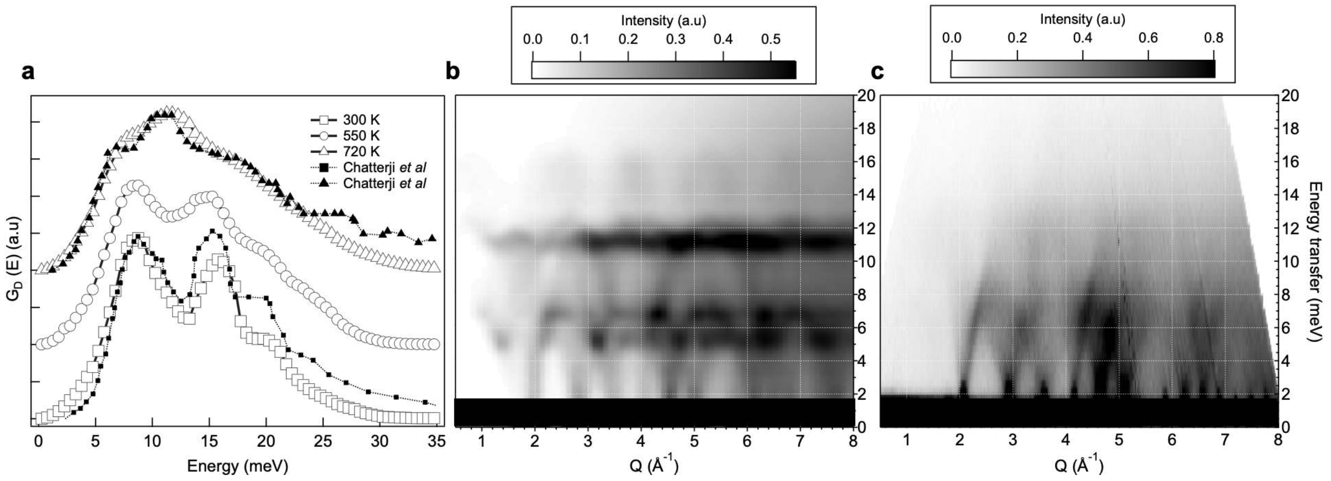

S 6| Phonon spectroscopy for GeTe.a, Experimental phonon density of states at various temperatures and comparison to literature data [30]. Note that data below ~2 meV were extrapolated using a parabolic fit due to elastic line contamination; **b,** Calculated powder averaged $S(Q,\omega)$ at 720 K using the harmonic dispersion described in the main text, note the band of intensity from the optic modes around 11.5 meV; Experimental $S(Q,\omega)$ measured at 720 K, using E_i = 40 meV neutrons. Note the massive broadening of the optic phonons, and the larger damping as a function of Q.

S7: Real-space thermal diffuse scattering in GeTe as a function of temperature.

As described in the **SI** and main body of the paper, c-GeTe is highly crystalline, although the sharp Bragg peaks co-exist with very strong diffuse scattering. Having shown that this signal is entirely inelastic, and results from strongly anharmonic phonons, logically:

- All deviations in the X-ray PDF from average structure reflect (inelastic) thermal diffuse scattering.
- This signal can be isolated by fitting the average structure at long-ranges (20-50 Å), then calculating the difference between observed and calculated PDF over the range 1.5 $<$ r $<$ 50 Å.
- At the local scale, the PDF contains anisotropic information, despite powder averaging. This is because of the high symmetry, and limited peak overlap below ~1 nm.

This is the central ansatz underlying our discovery of anisotropic elastic fluctuations in GeTe. In this section, we revisit the small-box fits to the X-ray PDFs of GeTe, and highlight the temperature dependence of this information. Firstly, recall our description of real-space correlated motion in sections 8.1 and 8.2. This is usually dealt with by refining an isotropic r-dependent sharpening parameter. Fig. **S7a** shows fits to GeTe in both phases, with two residuals (obs-calc) plotted. In both cases, the top residual reflects the best possible fit to the range 1.5-20 Å using the structures reported in section 1.4. This uses a standard isotropic sharpening parameter. The bottom residual in both cases represents the deviations from the average structure refined over the range 20-50 Å.
So what is unique about this data compared to e.g. the misfits seen when correlated motion sharpening is ignored for nickel (2)?

- Firstly, the unfitted sharpening of the <100>$_c$ peak shows that fluctuations are *anisotropic*. That is to say, the state-of-the-art models (section 8.2) for correlated motion do not work.

- Secondly, the inelastic diffuse scattering in GeTe is much stronger than in other simple cubic materials. This implies that GeTe is very soft, and anharmonic, even at room temperature.

All of these points are highlighted by the map of the real-space thermal diffuse scattering as a function of temperature shown in Fig. **S7b**. As employed in the main text, the height of these peaks is a model independent measure of elastic anisotropy in GeTe.

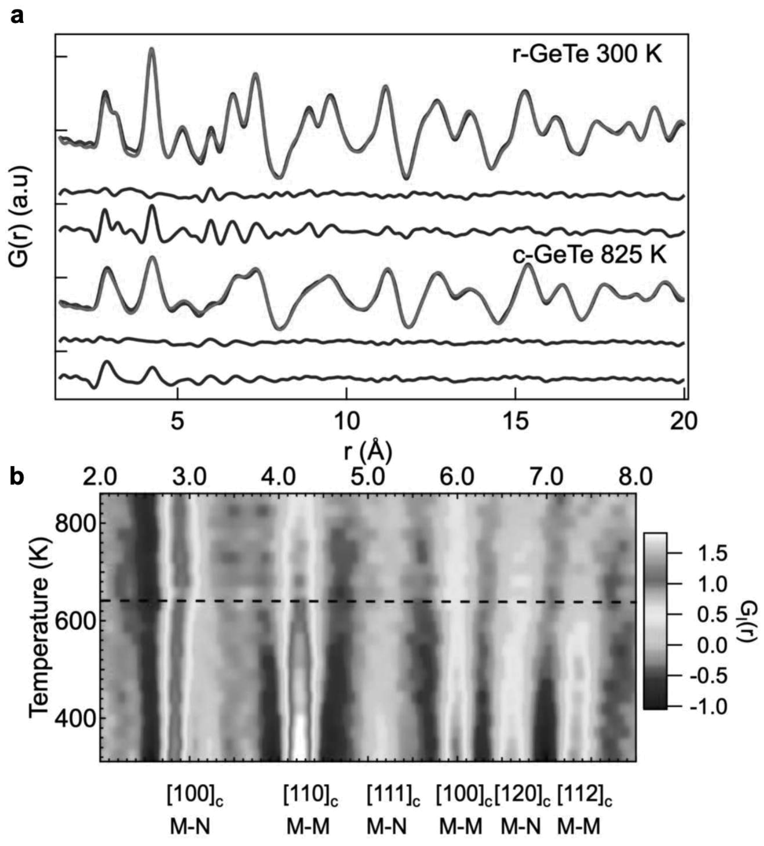

S 7| **Real-space thermal diffuse scattering in GeTe**. (top) Observed, calculated and difference fits to the X-ray PDFs of GeTe at 300 and 825 K. Two residual curves are shown for each fit, that arising from the fits discussed in the text, and the result of fitting the average-structure at 20-50 Å; (bottom) Colour map showing the raw residuals after fitting all the X-ray PDF data sets with the average structure over the range $20 < r < 50$ Å. Each residual was normalised by the fitted scale factor.

S8: Ubiquity of <100>$_c$ correlations in binary chalcogenides and hR6 structured materials.

The attribution of misfits in the PDF to correlated motion/phonons is one of the central results of our paper. In this part of the SI, we summarise: 8.1: The effect of correlated motion/phonons on the pair distribution function; 8.2: The state of the art for modelling correlated motion using 'small box' PDF refinements [10, 11]; 8.3: Published evidence for emergent anisotropy in other binary chalcogenides and hR6 structured materials [31–34]; Some of the figures included in this section are taken from published work by other authors, and permission to reproduce them has been received.

8.1 The effect of correlated motion/phonons on the pair distribution function

Correlated motion in crystals can arise from a number of sources, for example the atoms making up a molecule in a crystal obviously move together. Here we limit ourselves only to collective lattice vibrations in continuous network solids (phonons). These appear ubiquitously in diffraction experiments as a 'thermal diffuse' background underlying the Bragg peaks. This low-frequency signal obviously appears only at relatively low-r in the PDF (typically 1-1.5 nm).

The effect of including this signal on the radial distribution function (RDF) of nickel was described by McQueeney [10] [the relationship between the RDF and PDF used here can be found elsewhere [35]]. In the absence of collective dynamics, there are three contributions to the width of an experimental peak in the PDF. These are: a) The dispersion of atomic distances which contribute around distance r; b) The effect of random thermal motions, which gives a Gaussian broadening; and c) The width which comes from the finite experimental Q_{max} (i.e. the experimental resolution). The effect of adding in the thermal diffuse scattering is to sharpen peaks at low-r. This can be imagined as arising from correlated motion (i.e. the width of a peak between atoms moving in phase is reduced). The separate parts of the Ni radial distribution function are shown in **S8.1**. Here, the 'instantaneous' RDF measured in a standard experiment can be seen to be the sum of the average structure RDF and the inelastic RDF. This arises because the total neutron scattering cross section (see later) is simply the sum of the elastic and inelastic parts, i.e. $\sigma_{tot} = \sigma_{elas} + \sigma_{inelas}$. The inelastic part of the RDF appears as 'W' shaped features under the peaks, these redistribute intensity from the wings of the peaks to the centre, hence sharpening the peaks. Importantly, the same characteristic shape will be observed in the *residual* profile (i.e. observed-calculated difference) for any fit to a PDF which fails to account for dynamics (see next section).

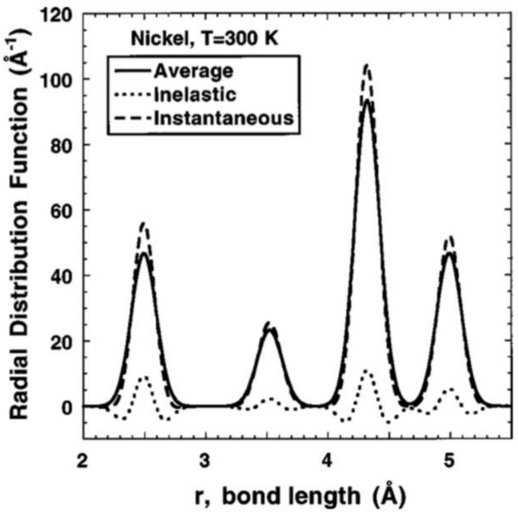

S8. 1| Effect of inelastic scattering on the radial distribution function of nickel. Reproduced with permission from McQueeney PRB **57** 10560 (1997). See: https://journals.aps.org/prb/abstract/10.1103/PhysRevB.57.10560.

8.2 The state of the art for modelling correlated motion using 'small box' PDF refinements

Within the small-box formalism for modelling PDFs, correlated motion from phonons is accounted for using sharpening parameters called `delta1` and `delta2`. These are derived from a simplified Debye model for the dynamics [11]. Below the Debye temperature of the sample, `delta1` is refined, and the correction falls off as $1/r$, as given by Eqn. 15. Here, the final calculated peak width is (σ_{ij}) for any atom pair (ij). The uncorrelated (long-range) peak width is σ'_{ij} and r_{ij} is the distance between atoms i and j.

$$\sigma_{ij} = \sigma'_{ij}\sqrt{(1 - \delta_1/r_{ij})}, \tag{15}$$

Above the Debye temperature, the length scale of correlated motion is expected to fall off more rapidly due to increased anharmonic interactions. In this case, `delta2` is refined, and the peak width expression becomes:

$$\sigma_{ij} = \sigma'_{ij}\sqrt{(1 - \delta_2/r_{ij}^2)}, \tag{16}$$

An experimental example which highlights the importance of these corrections for polycrystalline nickel is shown in Fig. **S8.2**. The results of refining the structural model without and with a `delta1` are shown. The latter fit can clearly be seen to be far superior.

Note that the correlated motion corrections described here have a major simplification, which is that they are entirely isotropic. The sharpening factor is identical for vectors between atoms in any direction. Of course, all materials show some elastic anisotropy. However, the effects of this are sufficiently small to be almost unobservable for fcc crystal structures like Ni [11]. The failure of models 15 and 16 for cubic materials like GeTe is therefore one of the major results of the present investigation.

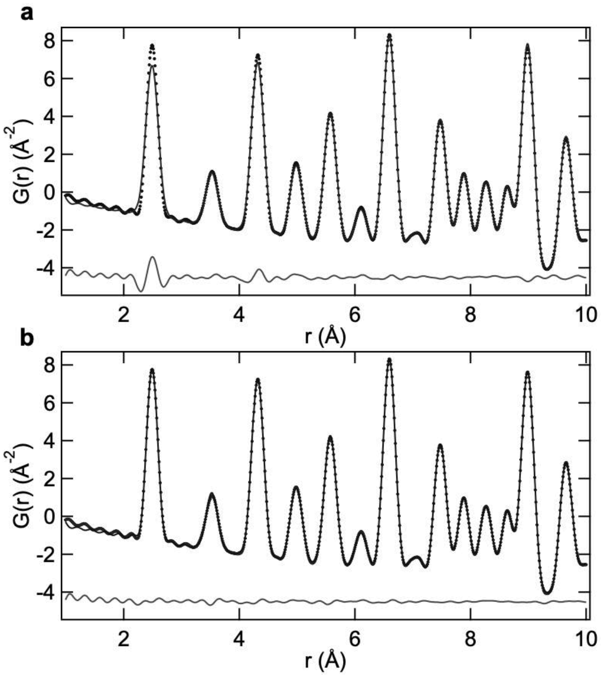

S8. 2| Effect of refining correlated motion corrections for nickel. Two fits to the same Ni X-ray PDF data set are shown here. These are: **a**: without refining the `delta1` parameter in PDFGui; **b**: After refining the delta1 parameter in PDFGui. The improvement to the fit is extremely notable at low-r, as judged by the characteristic 'W' shaped misfit in the residual in panel **a**.

8.3 Published evidence for emergent anisotropy in other binary chalcogenides and hR6 structured materials

As described in the main paper, the PDFs of GeTe shows anisotropic $<100>_c$ peak sharpening, which cannot be fitted using the isotropic expressions above. This is manifested as a misfit in the residual. The dynamic origin of this feature is confirmed by our vsPDF measurements. By examining published data for other binary chalcogenides and hR6 structured materials [31–34], we speculate that this is ubiquitous for this class of materials. Figure **S8.3** shows PDF fits for PbTe, SnTe, Bi and PbSe. The same characteristic misfit is seen for all samples, except at very low temperatures. This once again indicates that the $<100>_c$ sharpening originates from coupling to fluctuations.

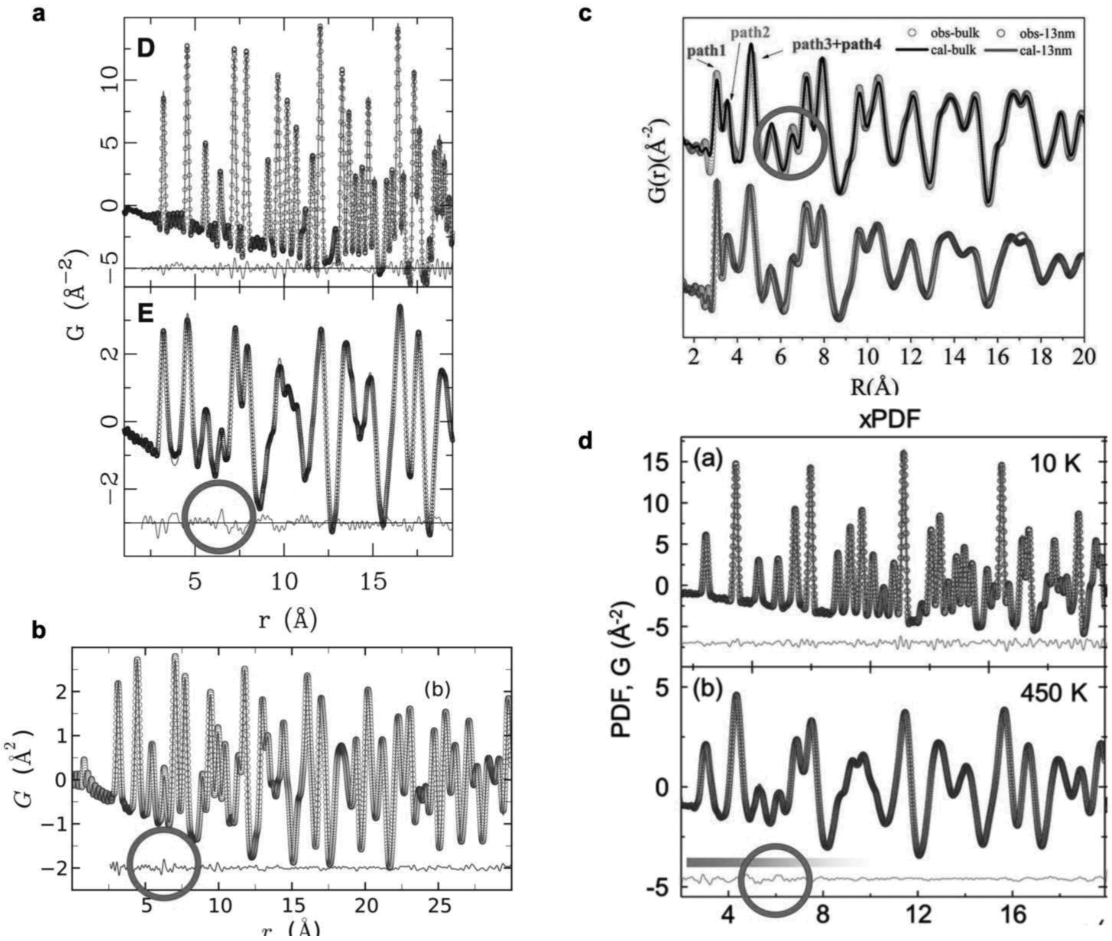

S8. 3| Ubiquity of <100>$_c$ correlations in binary chalcogenides and hR6 structured materials. This figure reproduces figures from published work. Red circles have been added to highlight the residual around the <100>$_c$ peaks. Permissions have been acquired as detailed for each panel, and full citations are included in the main manuscript text. **a,** Neutron PDFs for PbTe at 10 K (top) and 300 K (bottom). Fits to a rocksalt structure are shown. Figure reproduced with permission from Figure 1 of Bozin *et al*, Science, **330**, pp. 1660-1663 (2010). See: DOI: 10.1126/science.1192759. **b,** Fit of a rocksalt structure to the X-ray PDF of SnTe in the cubic phase. Figure reproduced with permission from Knox *et al*, PRB **89** 014102 (2014). See: https://journals.aps.org/prb/abstract/10.1103/PhysRevB.89.014102.**c,** X-ray PDF and fit for Bismuth metal (top curve). Figure reproduced under the terms of a creative commons license from Li *et al*, Advanced Science, **3**, 1600108 (2016). See: https://doi.org/10.1002/advs.201600108. **d,** Fit of a rocksalt structure to the X-ray PDF of PbSe in the cubic phase. Figure reproduced with permission from Yu *et al*, PRB **98** 144108 (2018). See:https://journals.aps.org/prb/abstract/10.1103/PhysRevB.98.144108.

S9: Extended theory results.

The purpose of this note is to derive an effective elastic coupling between polar degrees of freedom in a model for cubic ferroelectrics such as GeTe.

We consider a Ginzburg-Landau model with polarization $P_\alpha(\boldsymbol{x})$ ($\alpha = x, y, z$) coupled to the following elastic degrees of freedom as secondary order parameters: volumetric strain $\phi_v(\boldsymbol{x}) = (\epsilon_{xx} + \epsilon_{yy} + \epsilon_{zz})$, deviatoric stains $\phi_o(\boldsymbol{x}) = (\epsilon_{xx} - \epsilon_{yy})$, $\phi_t(\boldsymbol{x}) = (2\epsilon_{zz} - \epsilon_{xx} - \epsilon_{yy})/\sqrt{3}$, and shear strains $\phi_4(\boldsymbol{x}) = \epsilon_{yz}$, $\phi_5(\boldsymbol{x}) = \epsilon_{xz}$, and $\phi_6(\boldsymbol{x}) = \epsilon_{xy}$, where $\epsilon_{\alpha\beta} = (1/2)\left(\partial u_\alpha/\partial x_\beta + \partial u_\beta/\partial x_\alpha\right)$ is the linear strain tensor.

The Ginzburg-Landau Hamiltonian is as follows,

$$H = H_{\text{pol}} + H_{\text{elastic}} + H_{\text{pol-elastic}}, \tag{17}$$

where,

$$H_{\text{pol}} = \int d^3x \left[\frac{r}{2} \sum_\alpha P_\alpha^2(\boldsymbol{x}) + \sum_{\alpha,\beta} \left[u + v\delta_{\alpha\beta}\right] P_\alpha^2(\boldsymbol{x}) P_\beta^2(\boldsymbol{x}) + \frac{1}{2} \int d^3x' \sum_{\alpha\beta} F_{\alpha\beta}(\boldsymbol{x} - \boldsymbol{x}') P_\alpha(\boldsymbol{x}) P_\beta(\boldsymbol{x}') \right],$$

$$H_{\text{elastic}} = \int d^3x \left[\frac{1}{2} C_v \phi_v^2(\boldsymbol{x}) + \frac{1}{2} C_d \left[\phi_o^2(\boldsymbol{x}) + \phi_t^2(\boldsymbol{x})\right] + \frac{1}{2} C_s \left[\phi_4^2(\boldsymbol{x}) + \phi_5^2(\boldsymbol{x}) + \phi_6^2(\boldsymbol{x})\right] \right], \tag{18}$$

$$H_{\text{pol-elastic}} = \int d^3x g_s \left[\phi_4(\boldsymbol{x}) P_y(\boldsymbol{x}) P_z(\boldsymbol{x}) + \phi_5(\boldsymbol{x}) P_x(\boldsymbol{x}) P_z(\boldsymbol{x}) + \phi_6(\boldsymbol{x}) P_x(\boldsymbol{x}) P_y(\boldsymbol{x})\right]. \tag{19}$$

Here, $r = r_0(T - T_0)$, u and v are quartic temperature independent quartic aharmonicities, and $F_{\alpha\beta}(\boldsymbol{x}) = \left(x^2\delta_{\alpha\beta} - 3x_\alpha x_\beta\right)/x^3$ is the dipole interaction. C_v, C_d, and C_s are elastic modulli and g_s is an electrostrictive coupling constant to between polarization and shears. For our purposes (GeTe), we have assumed in (19) that the electrostrictive couplings to shears are dominant and ignore the couplings to volumetric and deviatoric strains.

We supplement the Hamiltonian (17) with the Saint-Venant condition [36],

$$\nabla \times (\nabla \times \boldsymbol{\epsilon})^T = 0, \tag{20}$$

which ensures compatibility between strains. In the absence of Eq. (20), the Hamiltonian (17) provides a standard long-wavelength description of the ferroelectric transition [37] in which the effect of the electrostrictive coupling is to generate a local quartic interaction between the polar degrees of freedom. By imposing compatibility, the coupling becomes non-local [38].

Assuming the system reaches mechanical equilibrium much faster than the polarization, we integrate out the strains in Eqs. (18) and (19) with the constraint (20) in favor of a quartic interaction between the polar degrees of freedom,

$$H_{\text{eff}} = -\frac{g_s^2}{2C_s} \int d^3q \sum_{\mu,\nu=4,5,6} K_{\mu\nu}(\hat{\boldsymbol{q}}) \Gamma_\mu(\boldsymbol{q}) \Gamma_\nu(-\boldsymbol{q}).$$

Here, $\Gamma_4(\boldsymbol{q})$, $\Gamma_5(\boldsymbol{q})$ and $\Gamma_6(\boldsymbol{q})$ are the Fourier components of $\Gamma_4(\boldsymbol{x}) = P_y(\boldsymbol{x})P_z(\boldsymbol{x})$, $\Gamma_5(\boldsymbol{x}) = P_x(\boldsymbol{x})P_z(\boldsymbol{x})$, and $\Gamma_6(\boldsymbol{x}) = P_x(\boldsymbol{x})P_y(\boldsymbol{x})$, respectively. The kernels $K_{\mu\nu}(\hat{\boldsymbol{q}})$ are non-analytic, e.g., consider $K_{66}(\hat{\boldsymbol{q}})$ at $q_z = 0$,

$$K_{66}(\hat{\boldsymbol{q}}) = \frac{\left(q_x^2 - q_y^2\right)^2 + (4/3)(C_t/C_v)\left(q_x^4 + q_y^4 + q_x^2 q_y^2\right)}{\left(q_x^2 - q_y^2\right)^2 + 4(C_t/C_s) q_x^2 q_y^2 + (4/3)(C_t/C_v)\left[q_x^4 + q_y^4 + q_x^2 q_y^2 (C_s + C_t)/C_s\right]}.$$

For $\boldsymbol{q} \parallel (110)$, $K_{66}(\hat{\boldsymbol{q}}) = C_s/(C_s + C_v + C_t/3)$, while $K_{66}(\hat{\boldsymbol{q}}) = 1$ for $\boldsymbol{q} \parallel (100)$. Away from $q_z = 0$, $K_{66}(\hat{\boldsymbol{q}})$ exhibits a butterfly pattern, as shown in Fig. 9. Non-analytic behavior is characteristic of elastic forces and is a consequence of their long-ranged and anisotropic nature [39]. Explicit expressions for $K_{\mu\nu}(\hat{\boldsymbol{q}})$ are very elaborate and are not provided here, nonetheless they also exhibit non-analytic behavior.

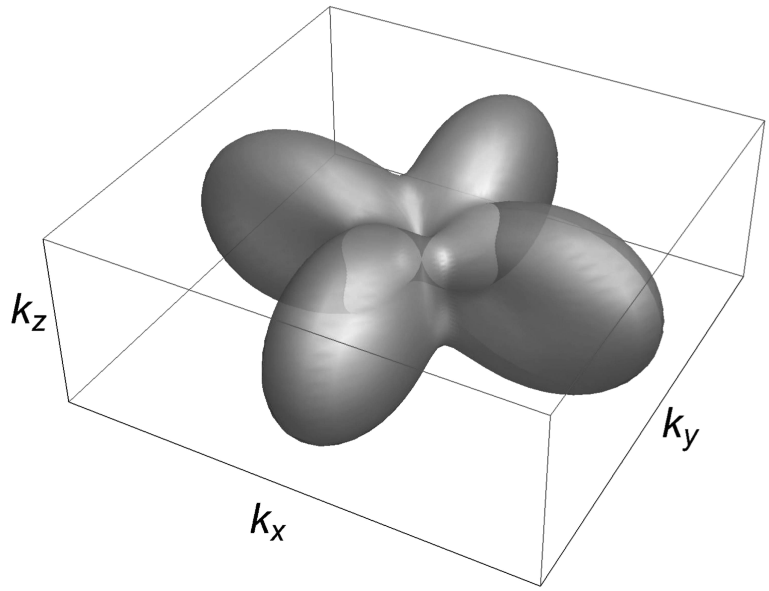

S 9| Wave-vector dependence of the strain-mediated coupling between polar degrees of freedom in the above model. The anisotropy is clear from the butterfly pattern.

References

1. Chatterji, T., Kumar, C. & Wdowik, U. D. Anomalous temperature-induced volume contraction in GeTe. *Physical Review B* **91,** 054110 (2015).
2. Chattopadhyay, T, Boucherle, J., *et al.* Neutron diffraction study on the structural phase transition in GeTe. *Journal of Physics C: Solid State Physics* **20,** 1431 (1987).
3. Levin, E., Besser, M. & Hanus, R. Electronic and thermal transport in GeTe: A versatile base for thermoelectric materials. *Journal of Applied Physics* **114,** 083713 (2013).
4. Sist, M., Kasai, H., Hedegaard, E. M. & Iversen, B. B. Role of vacancies in the high-temperature pseudodisplacive phase transition in GeTe. *Physical Review B* **97,** 094116 (2018).
5. Gainza, J. *et al.* Features of the high-temperature structural evolution of GeTe thermoelectric probed by neutron and synchrotron powder diffraction. *Metals* **10,** 48 (2019).
6. Kimber, S. A., Wildes, A. R., Mutka, H., Bos, J.-W. G. & Argyriou, D. N. Spin-chain correlations in the frustrated triangular lattice material CuMnO2. *Journal of Physics: Condensed Matter* **32,** 445802 (2020).
7. Egami, T. & Billinge, S. J. *Underneath the Bragg peaks: structural analysis of complex materials* (Newnes, 2012).
8. Farrow, C. *et al.* PDFfit2 and PDFgui: computer programs for studying nanostructure in crystals. *Journal of Physics: Condensed Matter* **19,** 335219 (2007).
9. Toby, B. & Egami, T. Accuracy of pair distribution function analysis applied to crystalline and non-crystalline materials. *Acta Crystallographica Section A: Foundations of Crystallography* **48,** 336–346 (1992).
10. McQueeney, R. Dynamic radial distribution function from inelastic neutron scattering. *Physical Review B* **57,** 10560 (1998).
11. Jeong, I.-K., Heffner, R., Graf, M. & Billinge, S. Lattice dynamics and correlated atomic motion from the atomic pair distribution function. *Physical Review B* **67,** 104301 (2003).
12. Warren, B. E. *X-ray Diffraction* (Courier Corporation, 1990).
13. Honkimäki, V & Suortti, P. Whole-pattern fitting in energy-dispersive powder diffraction. *Journal of applied crystallography* **25,** 97–104 (1992).
14. Squires, G. L. *Introduction to the theory of thermal neutron scattering* (Courier Corporation, 1996).

15. Abernathy, D. L. *et al.* Design and operation of the wide angular-range chopper spectrometer ARCS at the Spallation Neutron Source. *Review of Scientific Instruments* **83,** 015114 (2012).

16. Niedziela, J. L. *et al.* Design and operating characteristic of a vacuum furnace for time-of-flight inelastic neutron scattering measurements. *Review of Scientific Instruments* **88,** 105116 (2017).

17. Stone, M. B., Niedziela, J. L., Loguillo, M. J., Overbay, M. A. & Abernathy, D. L. A radial collimator for a time-of-flight neutron spectrometer. *Review of Scientific Instruments* **85,** 085101 (2014).

18. Paalman, H. H. & Pings, C. J. Numerical Evaluation of X□Ray Absorption Factors for Cylindrical Samples and Annular Sample Cells. *Journal of Applied Physics* **33,** 2635–2639 (1962).

19. Arnold, O. *et al.* Mantid—Data analysis and visualization package for neutron scattering and μ SR experiments. *Nuclear instruments and methods in physics research section a: accelerators, spectrometers, detectors and associated equipment* **764,** 156–166 (2014).

20. Hannon, A. C., Arai, M. & Delaplane, R. G. A dynamic correlation function from inelastic neutron scattering data. *Nuclear Instruments and Methods in Physics Research Section A: Accelerators, Spectrometers, Detectors and Associated Equipment* **354,** 96–103 (1995).

21. Egami, T. & Dmowski, W. Dynamic pair-density function method for neutron and x-ray inelastic scattering. *Zeitschrift für Kristallographie Crystalline Materials* **227,** 233–237 (2012).

22. Iwashita, T. *et al.* Seeing real-space dynamics of liquid water through inelastic x-ray scattering. *Science advances* **3,** e1603079 (2017).

23. Juhás, P., Louwen, J. N., Eijck, L. v., Vogt, E. T. & Billinge, S. J. PDFgetN3: atomic pair distribution functions from neutron powder diffraction data using ad hoc corrections. *Journal of Applied Crystallography* **51,** 1492–1497 (2018).

24. Loong, C.-K., Ikeda, S & Carpenter, J. The resolution function of a pulsed-source neutron chopper spectrometer. *Nuclear Instruments and Methods in Physics Research Section A: Accelerators, Spectrometers, Detectors and Associated Equipment* **260,** 381–402 (1987).

25. Chapman, K. W., Lapidus, S. H. & Chupas, P. J. Applications of principal component analysis to pair distribution function data. *Journal of Applied Crystallography* **48,** 1619–1626 (2015).

26. Butala, M. M. *et al.* Local structure evolution and modes of charge storage in secondary Li–FeS2 cells. *Chemistry of Materials* **29,** 3070–3082 (2017).

27. Liu, C.-H. *et al.* Validation of non-negative matrix factorization for rapid assessment of large sets of atomic pair distribution function data. *Journal of Applied Crystallography* **54,** 768–775 (2021).

28. Geddes, H. S., Hutchinson, H. D., Ha, A. R., Funnell, N. P. & Goodwin, A. L. Extracting interface correlations from the pair distribution function of composite materials. *Nanoscale* **13,** 13220–13224 (2021).

29. Islam, F. *et al.* Super-resolution energy spectra from neutron direct-geometry spectrometers. *Review of Scientific Instruments* **90,** 105109 (2019).

30. Chatterji, T, Rols, S & Wdowik, U. Dynamics of the phase-change material GeTe across the structural phase transition. *Frontiers of Physics* **14,** 23601 (2019).

31. Božin, E. S. *et al.* Entropically stabilized local dipole formation in lead chalcogenides. *Science* **330,** 1660–1663 (2010).

32. Knox, K., Bozin, E., Malliakas, C., Kanatzidis, M. G. & Billinge, S. Local off-centering symmetry breaking in the high-temperature regime of SnTe. *Physical Review B* **89,** 014102 (2014).

33. Li, Q. *et al.* Local structural distortion induced uniaxial negative thermal expansion in nanosized semimetal bismuth. *Advanced Science* **3,** 1600108 (2016).

34. Yu, R. *et al.* Emphanitic anharmonicity in PbSe at high temperature and anomalous electronic properties in the Pb Q (Q= S, Se, Te) system. *Physical Review B* **98,** 144108 (2018).

35. Keen, D. A. A comparison of various commonly used correlation functions for describing total scattering. *Journal of Applied Crystallography* **34,** 172–177 (2001).

36. Oliver, X. & Agelet de Saracibar, C. *Continuum Mechanics for Engineers. Theory and Problems* (2nd edition, 2017).

37. Cowley, R. A. Structural phase transitions I. Landau theory. *Adv. Phys.* **29,** 1 (1980).

38. Brierley, R. T. & Littlewood, P. B. Domain wall fluctuations in ferroelectrics coupled to strain. *Phys. Rev. B* **89,** 184104 (2014).

39. Bratkovsky, A., Marais, S., Heine, V & Salje, E. The theory of fluctuations and texture embryos in structural phase transitions mediated by strain. *J. Phys. Condens. Matter* **6,** 3679 (1994).
